# Correction: Carbon nanotube–vitrimer composite for facile and efficient photo-welding of epoxy

**DOI:** 10.1039/c6sc90083f

**Published:** 2017-01-25

**Authors:** Yang Yang, Zhiqiang Pei, Xiqi Zhang, Lei Tao, Yen Wei, Yan Ji

**Affiliations:** a The Key Laboratory of Bioorganic Phosphorus Chemistry & Chemical Biology , Department of Chemistry , Tsinghua University , Beijing 100084 , China . Email: jiyan@mail.tsinghua.edu.cn

## Abstract

Correction for ‘Carbon nanotube–vitrimer composite for facile and efficient photo-welding of epoxy’ by Yang Yang *et al.*, *Chem. Sci.*, 2014, **5**, 3486–3492.



## 

Throughout the original manuscript, incorrect values were given for the light intensity for the IR laser irradiation. All instances of “15.2 W cm^–2^” should be changed to “0.82 W cm^–2^”, and all instances of “1.52 W cm^–2^” should be changed to “0.25 W cm^–2^”.

The Royal Society of Chemistry apologises for these errors and any consequent inconvenience to authors and readers.

